# Vermeidbare Sterblichkeit – Neufassung eines Indikators für die Präventionsberichterstattung

**DOI:** 10.1007/s00103-021-03458-y

**Published:** 2021-12-08

**Authors:** Anke Weber, Veronika Reisig, Andrea Buschner, Joseph Kuhn

**Affiliations:** 1 Hochschule Hamm-Lippstadt, Marker Allee 76–78, 59063 Hamm, Deutschland; 2 Bayerisches Landesamt für Gesundheit und Lebensmittelsicherheit, Oberschleißheim, Deutschland; 3 Bayerisches Landesamt für Statistik, Fürth, Deutschland

**Keywords:** Vermeidbare Sterbefälle, OECD-Eurostat Liste, Gesundheitsberichtserstattung, Todesursachenstatistik, Prävention, Avoidable mortality, OECD-Eurostat list, Health reporting, Causes of death statistics, Prevention

## Abstract

**Hintergrund:**

In der Gesundheitsberichterstattung wird ein Indikator „vermeidbare Sterblichkeit“ geführt. Der Indikator aggregiert ausgewählte Todesursachen. In Deutschland gibt es dazu 2 Varianten, beide sind nicht mehr aktuell. Mit der vorliegenden Arbeit wird eine Neukonzeption vorgeschlagen.

**Methoden:**

Die Neukonzeption orientiert sich bei der Auswahl der Todesursachen an Vorarbeiten auf europäischer Ebene. Die Umsetzbarkeit und Plausibilität einer konsentierten OECD-Eurostat-Liste werden anhand der Daten der amtlichen Statistik in Bayern für die Jahre 2016–2018 überprüft. Die Analyse umfasst die Untersuchung der Variabilität über die Zeit und innerhalb der bayerischen Regierungsbezirke sowie mögliche systematische Verzerrungen durch regionale Unterschiede im Codierverhalten bzw. Veränderungen im Zeitverlauf.

**Ergebnisse:**

Die OECD-Eurostat-Liste ist auf regionaler Ebene mit geringfügiger Modifikation umsetzbar. Es ergibt sich eine altersstandardisierte vermeidbare Sterblichkeit von knapp 23 Todesfällen je 10.000 Einwohnerinnen und Einwohner (EW) in Bayern im Jahr 2018, wobei die prävenierbaren Sterbefälle solche, die durch eine Behandlung hätten vermieden werden können, bei Weitem übersteigen. Für Männer liegt die Sterberate aufgrund vermeidbarer Ursachen bei 30 pro 10.000 männlichen EW und ist damit fast doppelt so hoch wie jene für Frauen (16 pro 10.000 weiblichen EW). Regional folgt die vermeidbare Sterblichkeit Befunden zur regionalen Gesundheit aus anderen Studien.

**Diskussion und Fazit:**

Die Ergebnisse liefern keinen Anlass, von großen Zufallsschwankungen bzw. methodisch bedingten systematischen Verzerrungen auszugehen. Der Indikator wird zur Anwendung in der Gesundheitsberichterstattung vorgeschlagen.

## Abstract (en)

**Background:**

“Avoidable mortality” is used as an indicator in health reporting. The indicator aggregates selected causes of death. In Germany two versions exist, both of which are no longer up to date. A new version is proposed.

**Methods:**

The new version is based on preparatory work on the European level. The feasibility and plausibility of a common OECD Eurostat list are examined using official statistics data from Bavaria from 2016 to 2018. The analysis includes an examination of the variability over time and within the Bavarian administrative districts, as well as possible systematic errors through regional differences in coding behaviour or changes over time.

**Results:**

The OECD Eurostat list can be implemented at the regional level with only minor modifications. The age-standardized avoidable mortality in Bavaria in 2018 is almost 23 deaths per 10,000 inhabitants, with preventable deaths by far outweighing treatable deaths. For men, the death rate due to avoidable causes is 30 per 10,000 male inhabitants, which is almost twice as high as that for women (16 per 10,000 female inhabitants). The regional findings on avoidable mortality are consistent with findings on regional health from other studies.

**Discussion and conclusion:**

The results do not indicate a threat to reliability due to random fluctuation or systematic methodological errors. The new version is recommended for application in health reporting.

## Hintergrund

Die Gesundheitsberichterstattung stellt Daten zur gesundheitlichen Lage der Bevölkerung bereit, einschließlich Daten zu Gesundheitsdeterminanten und Daten zur Gesundheitsversorgung. Aspekte der Prävention spielen dabei eine wichtige Rolle. Die Gesundheitsdienstgesetze der Länder sehen für die Gesundheitsberichterstattung auf Landesebene meist eine explizite Orientierung auf die Prävention und Gesundheitsförderung vor [1]. Durch die Verabschiedung des Präventionsgesetzes (PrävG 2015) im Jahr 2015, das in seiner Umsetzung auch der Länderebene eine wichtige Rolle zuweist, wurde diese präventive Akzentuierung weiter gestärkt.

Das Präventionsgesetz verpflichtet die Nationale Präventionskonferenz, dem Bundesministerium für Gesundheit alle 4 Jahre einen Präventionsbericht zuzuleiten. Die zur Umsetzung des Gesetzes in den Ländern geschlossenen Landesrahmenvereinbarungen nach § 20f des Fünften Buches Sozialgesetzbuch (SGB V) nehmen ebenfalls explizit Bezug auf die Gesundheitsberichterstattung. Dadurch angestoßen beschäftigen sich Akteure auf Bundes- wie Länderebene mit der Konzeptualisierung und den Entwicklungsmöglichkeiten einer Präventionsberichterstattung als Datengrundlage für Aktivitäten auf den unterschiedlichen regionalen Ebenen [1–4]. Hierbei spielen auch methodische Aspekte, darunter die Entwicklung bzw. Weiterentwicklung relevanter Indikatoren, eine wichtige Rolle [5].

Ein Indikator, der sowohl Aspekte der Gesundheitsversorgung wie auch der Prävention abbildet und seit Längerem in der Gesundheitsberichterstattung Anwendung findet, ist die vermeidbare Sterblichkeit. Der Indikator ist ein Maß für die Qualität des Gesundheitssystems mit Blick darauf, in welchem Umfang Sterbefälle auftreten, die bei angemessener Prävention bzw. Therapie in einer bestimmten Altersgruppe grundsätzlich hätten verhindert werden können. Zugrunde gelegt wird dabei eine Auswahl von Todesursachen, die als sensibel für Effekte der Prävention und Versorgung gelten.

### Internationale Entwicklung der Konzepte zur Messung vermeidbarer Sterblichkeit

Das Konzept „vermeidbare Sterblichkeit“ wurde von Rutstein et al. [6] entwickelt. Während ursprünglich die Qualität der medizinischen Versorgung im Vordergrund stand, bezogen weiterentwickelte Ansätze verstärkt auch Präventionsaspekte ein. Wiederholt wurde die zugrunde liegende Liste von Todesursachen und Altersgrenzen an die aktuellen medizinischen Standards angepasst. Hier sind insbesondere die Arbeiten von Holland [7–9] sowie von Nolte und McKee [10, 11] und das AMIEHS-Projekt der Europäischen Union (EU) („amenable mortality in the European Union“, [12]) zu nennen.

Basierend auf diesen Vorarbeiten entwickelte eine Arbeitsgruppe auf Anfrage der europäischen Mitgliedsstaaten im Jahr 2015 eine für die europäischen Mitgliedsstaaten einheitliche Liste der vermeidbaren Sterblichkeit, die in großen Teilen auf der Zusammenstellung des Office for National Statistics (ONS) im Vereinigten Königreich aus dem Jahr 2011 beruht [13]. Die vermeidbare Sterblichkeit wurde zudem von der Europäischen Kommission als Indikator für die Leistung des Gesundheitssystems eines Landes in den Gemeinsamen Bewertungsrahmen für Gesundheit aufgenommen [14]. Im Jahr 2018 wurde des Weiteren in enger Zusammenarbeit mit der Arbeitsgruppe für vermeidbare Sterblichkeit der Organisation für wirtschaftliche Zusammenarbeit und Entwicklung (OECD) eine kohärente Liste für europäische und OECD-Länder entwickelt, die von den zuständigen Arbeitsgruppen für Gesundheitsstatistik der OECD und von Eurostat im Jahr 2018 genehmigt [15] und im darauffolgenden Jahr nochmals geringfügig modifiziert wurde.

Die finale OECD-Eurostat-Liste [15] stellt somit die einzige von allen europäischen und OECD-Ländern auf internationaler Ebene anerkannte Liste der vermeidbaren Sterblichkeit dar. Sie umfasst zudem eine explizite Zuschreibung der vermeidbaren Todesursachen in *prävenierbare Todesursachen* und *behandelbare Todesursachen*. Dabei beziehen sich prävenierbare Todesursachen auf Krankheiten oder Unfälle, die durch geeignete Maßnahmen der Primärprävention gar nicht aufgetreten wären, und behandelbare Todesursachen auf Fälle, welche durch rechtzeitige und wirksame Gesundheitsmaßnahmen, einschließlich sekundärer Prävention und Behandlung, vermeidbar gewesen wären [15]. Die Zuordnung zur jeweiligen Kategorie erfolgt danach, ob die Todesursache vorwiegend durch Präventions- oder Gesundheitsinterventionen hätte vermieden werden können. Bei einigen Todesursachen konnte diese vorwiegende Zuordnung nicht erfolgen, dort wurden die Todesfälle zu 50 % als prävenierbar und zu 50 % als durch Behandlung vermeidbar eingestuft.

In der OECD-Eurostat-Liste werden Sterbefälle ab 0 Jahren bis unter 75 Jahren einbezogen. Diese Altersgrenze basiert auf der aktuellen Lebenserwartung in europäischen und entwickelten Ländern und spiegelt das Ausmaß wider, in dem ein Tod unter 75 Jahren mit hoher Wahrscheinlichkeit hätte verhindert werden können.

### Messung vermeidbarer Sterblichkeit in Deutschland

In Deutschland hat die methodische Weiterentwicklung des Indikators der vermeidbaren Sterblichkeit wenig Aufmerksamkeit erfahren [16]. Unbenommen der internationalen Entwicklung werden hier zurzeit 2 unterschiedliche Todesursachenlisten für die vermeidbare Sterblichkeit verwendet [17]: eine basierend auf Vorschlägen des Sachverständigenrates zur Begutachtung der Entwicklung im Gesundheitswesen (SVR Gesundheit), die sich an die Definition von Charlton und Velez aus dem Jahr 1986 anlehnt [18], und eine Liste aus dem Indikatorensatz der Arbeitsgemeinschaft der Obersten Landesgesundheitsbehörden (AOLG) für die Gesundheitsberichterstattung der Länder [19]. Während der Indikator des SVR Gesundheit, der in der Gesundheitsberichterstattung auf Bundesebene benutzt wird, sich weniger für eine Darstellung auf regionaler Ebene eignet [20], soll der Indikator der AOLG in der Lage sein, vermeidbare Sterblichkeit regional zu messen [17]. Beide Listen definieren im Vergleich zur OECD-Eurostat-Liste nur sehr wenige Todesursachen als vermeidbar^1^, differenzieren nicht nach behandelbaren bzw. prävenierbaren Todesursachen und nutzen unterschiedliche Altersbereiche, meist mit einer Obergrenze von unter 65 Jahren.

Hinsichtlich des Fortschritts in Gesundheitsversorgung und Prävention, der konzeptionellen Weiterentwicklungen auf internationaler Ebene und des Bedarfs einer wissenschaftlich fundierten Datengrundlage für Bund und Länder ergibt sich daher ein Überarbeitungsbedarf der in Deutschland genutzten Indikatoren der vermeidbaren Sterblichkeit. Ein neu konzipierter Indikator sollte:

1. eine an die aktuellen medizinischen, präventiven und demografischen Entwicklungen angepasste Auswahl von Todesursachen und Altersgrenzen enthalten,
2. den prävenierbaren und behandelbaren Anteil der vermeidbaren Sterblichkeit sichtbar machen
3. und nach Möglichkeit für eine regionale Anwendung geeignet sein.

Im vorliegenden Artikel wird eine solche Neukonzeption vorgeschlagen sowie eine empirische Überprüfung des Indikators für Bayern und seine Regierungsbezirke unternommen.

## Methoden

Basis der Neufassung ist die OECD-Eurostat-Liste zu vermeidbaren Sterbefällen aus dem Jahr 2019, da diese eine Differenzierung von Todesfällen in „prävenierbar“ und „durch Behandlung vermeidbar“ enthält und den aktuellen Stand medizinischer wie präventiver Möglichkeiten sowie den Konsens auf europäischer Ebene widerspiegelt.

### Anpassungen der OECD-Eurostat-Liste

#### Todesursachenkategorien.

Für die vorliegende Arbeit werden alle in der OECD-Eurostat-Liste enthaltenen Todesursachen zugrunde gelegt (Tab. 1), zum einen aus Gründen der besseren (internationalen) Vergleichbarkeit, zum anderen aufgrund des für jede Todesursache ausgewiesenen Potenzials, durch Prävention oder Behandlung vermieden zu werden [15].

**Table 1:** 

Todesursache	ICD-10-Ziffern	Einbezug in Berechnung der prävenierbaren Sterblichkeit	Einbezug in Berechnung der durch Behandlung vermeidbaren Sterblichkeit
Infektiöse Darmkrankheiten	A00–A09	Ja	Nein
Diphtherie, Tetanus, Poliomyelitis	A35, A36, A80	Ja	Nein
Keuchhusten	A37	Ja	Nein
Meningokokkeninfektion	A39	Ja	Nein
Sepsis durch Pneumokokken, Sepsis durch Haemophilus influenzae	A40.3, A41.3	Ja	Nein
Infektion durch *Haemophilus influenzae*	A49.2	Ja	Nein
Sexuell übertragbare Infektionen (außer HIV/Aids)	A50–A60, A63, A64	Ja	Nein
Varizellen (Windpocken)	B01	Ja	Nein
Masern	B05	Ja	Nein
Röteln	B06	Ja	Nein
Virushepatitis	B15–B19	Ja	Nein
HIV/Aids	B20–B24	Ja	Nein
Malaria	B50–B54	Ja	Nein
Meningitis durch *Haemophilus influenzae* und Pneumokokken	G00.0, G00.1	Ja	Nein
Tuberkulose	A15–A19^a^, B90^a^, J65	Ja (50 %)	Ja (50 %)
Scharlach	A38	Nein	Ja
Streptokokkensepsis, sonstige Sepsis	A40 (außer A40.3), A41 (außer A41.3)	Nein	Ja
Erysipel (Wundrose), Phlegmone	A46, L03	Nein	Ja
Legionellose mit Pneumonie	A48.1	Nein	Ja
Streptokokken- und Enterokokkeninfektion	A49.1	Nein	Ja
Andere Meningitis	G00.2, G00.3, G00.8, G00.9	Nein	Ja
Meningitis durch sonstige und nicht näher bezeichnete Ursachen	G03	Nein	Ja
Bösartige Neubildungen der Lippe, der Mundhöhle und des Pharynx	C00–C14	Ja	Nein
Bösartige Neubildung des Ösophagus	C15	Ja	Nein
Bösartige Neubildung des Magens	C16	Ja	Nein
Bösartige Neubildung der Leber und der intrahepatischen Gallengänge	C22	Ja	Nein
Bösartige Neubildung der Trachea, der Bronchien und der Lunge	C33–C34^b^	Ja	Nein
Mesotheliom	C45	Ja	Nein
Bösartiges Melanom der Haut	C43	Ja	Nein
Bösartige Neubildung der Harnblase	C67	Ja	Nein
Bösartige Neubildung der Cervix uteri	C53^a,b^	Ja (50 %)	Ja (50 %)
Bösartige Neubildung des Kolons, am Rektosigmoid, des Rektums und des Anus	C18–C21	Nein	Ja
Bösartige Neubildungen der Brustdrüse (Mamma)	C50^b^	Nein	Ja
Bösartige Neubildung des Corpus uteri	C54–C55	Nein	Ja
Bösartige Neubildung des Hodens	C62	Nein	Ja
Bösartige Neubildung der Schilddrüse	C73	Nein	Ja
Hodgkin-Lymphom (Lymphogranulomatose)	C81^a^	Nein	Ja
Lymphatische Leukämie	C91.0, C91.1	Nein	Ja
Gutartige Neubildungen	D10–D36	Nein	Ja
Alimentäre Anämien	D50–D53	Ja	Nein
Diabetes mellitus	E10–E14	Ja (50 %)	Ja (50 %)
Krankheiten der Schilddrüse	E00–E07	Nein	Ja
Nebennierenerkrankung	E24–E25 (außer E24.4), E27	Nein	Ja
Epilepsie	G40, G41	Nein	Ja
Aortenaneurysma und -dissektion	I71	Ja (50 %)	Ja (50 %)
Hypertonie (Hochdruckkrankheit)	I10–I13^a,b^, I15^a,b^	Ja (50 %)	Ja (50 %)
Ischämische Herzkrankheiten	I20–I25^b^	Ja (50 %)	Ja (50 %)
Zerebrovaskuläre Krankheiten	I60–I69^a,b^	Ja (50 %)	Ja (50 %)
Atherosklerose und nicht näher bezeichnete periphere Gefäßkrankheit	I70, I73.9	Ja (50 %)	Ja (50 %)
Rheumatische und andere Herzkrankheiten	I00–I09^a^	Nein	Ja
Venöse Thromboembolien	I26, I80, I.82.9	Nein	Ja
Grippe	J09–J11	Ja	Nein
Pneumonie durch *Streptococcus pneumoniae* oder *Haemophilus influenzae*	J13–J14	Ja	Nein
Chronische Bronchitis	J40–J44	Ja	Nein
Lungenkrankheiten durch exogene Substanzen	J60–J64, J66–J70, J82, J92	Ja	Nein
Infektionen der oberen Atemwege	J00–J06, J30–J39	Nein	Ja
Pneumonie, nicht näher bezeichnet oder Erreger nicht näher bezeichnet	J12, J15, J16–J18	Nein	Ja
Akute Infektionen der unteren Atemwege	J20–J22	Nein	Ja
Asthma und Bronchiektasen	J45–J47	Nein	Ja
Atemnotsyndrom bei Erwachsenen	J80	Nein	Ja
Lungenödem	J81	Nein	Ja
Abszess der Lunge und des Mediastinums, Pyothorax	J85, J86	Nein	Ja
Sonstige Krankheiten der Pleura	J90, J93, J94	Nein	Ja
Magen- und Zwölffingerdarmgeschwür	K25–K28	Nein	Ja
Krankheiten der Appendix	K35–K38^a^	Nein	Ja
Hernien	K40–K46	Nein	Ja
Cholelithiasis und Cholezystitis	K80–K81^a^	Nein	Ja
Sonstige Krankheiten der Gallenblase und der Gallenwege	K82–K83^a^	Nein	Ja
Akute Pankreatitis	K85.0,1,3,8,9	Nein	Ja
Sonstige Krankheiten des Pankreas	K86.1,2,3,8,9	Nein	Ja
Nephritisches und nephrotisches Syndrom	N00–N07	Nein	Ja
Obstruktive Uropathie	N13, N20–N21, N35	Nein	Ja
Niereninsuffizienz	N17–N19	Nein	Ja
Nierenkolik	N23	Nein	Ja
Krankheiten infolge Schädigung der tubulären Nierenfunktion	N25	Nein	Ja
Schrumpfniere, nicht näher bezeichnet, kleine Niere unbekannter Ursache	N26–N27	Nein	Ja
Entzündliche Erkrankungen des Urogenitalsystems	N34.1, N70–N73, N75.0, N75.1, N76.4, N76.6	Nein	Ja
Prostatahyperplasie	N40	Nein	Ja
Tetanus neonatorum	A33	Ja	Nein
Tetanus während der Schwangerschaft, der Geburt und des Wochenbettes	A34	Ja	Nein
Schwangerschaft, Geburt und Wochenbett	O00–O99^a^	Nein	Ja
Bestimmte Zustände, die ihren Ursprung in der Perinatalperiode haben	P00–P96	Nein	Ja
Bestimmte angeborene Fehlbildungen des Nervensystems	Q00, Q01, Q05	Ja	Nein
Angeborene Fehlbildungen des Kreislaufsystems	Q20–Q28	Nein	Ja
Medikamente, Arzneimittel und biologische Substanzen, die bei der therapeutischen Verwendung unerwünschte Wirkungen verursachen	Y40–Y59	Nein	Ja
Komplikationen bei der medizinischen und chirurgischen Behandlung	Y60–Y69, Y83–Y84	Nein	Ja
Medizinische Produkte im Zusammenhang mit unerwünschten Nebenwirkungen bei der Diagnose und therapeutischen Anwendung	Y70–Y82	Nein	Ja
Transportmittelunfälle	V01–V99^b^	Ja	Nein
Unfallverletzungen	W00–X39, X46–59	Ja	Nein
Absichtliche Selbstverletzung	X66–X84	Ja	Nein
Ereignis, dessen nähere Umstände unbestimmt sind	Y16–Y34	Ja	Nein
Tätlicher Angriff	X86–Y09	Ja	Nein
Alkoholbedingte Krankheiten: alkoholspezifische Störungen und Vergiftungen	E24.4, F10, G31.2, G62.1, G72.1, I42.6, K29.2, K70, K85.2, K86.0, Q86.0, R78.0, X45, X65, Y15	Ja	Nein
Alkoholbedingte Krankheiten: andere alkoholbedingte Störungen	K73, K74.0–K74.2, K74.6	Ja	Nein
Erkrankungen durch Drogenkonsum: drogenbedingte Störungen und Vergiftung	F11–F16, F18–F19, X40–X44, X85, Y10–Y14	Ja	Nein
Erkrankungen durch Drogenkonsum: absichtliche Selbstvergiftung durch Drogen	X60–X64	Ja	Nein

„Ja (50 %)“ bedeutet, dass 50 % der Sterbefälle dieser Todesursachenkategorie in die Berechnung für die genannte Sterblichkeit einbezogen werden

*ICD* Internationale Klassifikation der Krankheiten

^a^Auch in der Liste des Sachverständigenrates zur Begutachtung der Entwicklung im Gesundheitswesen (SVR Gesundheit) enthalten, wenngleich mit anderen Altersgrenzen (von I00–I09 sind nur die Codes I05–I09 in der SVR-Liste enthalten)

^b^Auch in der Liste der Arbeitsgemeinschaft der Obersten Landesgesundheitsbehörden (AOLG) enthalten, wenngleich mit anderen Altersgrenzen

#### Altersstandardisierung.

Um den Einfluss unterschiedlicher Altersstrukturen der verglichenen Populationen auf den Indikator auszuschließen, wird der Indikator altersstandardisiert. Während bei internationalen Berechnungen häufig die Europa-Standardbevölkerung bzw. die OECD-Standardbevölkerung benutzt wird, ist für einen innerdeutschen Vergleich eine auf Deutschland angepasste Standardbevölkerung zweckmäßiger. Für die vorliegende Untersuchung wird eine direkte Altersstandardisierung mit der Standardbevölkerung „Deutschland 2011“ vorgenommen. Um einen Vergleich des Indikators für Männer und Frauen zu gewährleisten, wird dabei mit der Gesamtbevölkerung gewichtet. Für internationale Vergleiche kann der Indikator ohne Probleme mit anderen Standardbevölkerungen gewichtet werden.

#### Altersgrenze.

Die OECD-Eurostat-Liste nutzt eine Altersgrenze von 75 Jahren zur Bestimmung eines vermeidbaren vorzeitigen Todes, d. h., einbezogen werden Todesfälle der ausgewählten Todesursachen in einem Alter unter 75 Jahren. Die Altersgrenze von 75 Jahren liegt wesentlich höher als die im SVR-Gesundheit- bzw. AOLG-Indikator verwendeten Grenzen und trägt der gestiegenen Lebenserwartung in Europa Rechnung. Sie spiegelt das Ausmaß wider, in dem ein Tod vor dem Alter von 75 Jahren mit hoher Wahrscheinlichkeit hätte verhindert werden können. Hier wird daher die Altersgrenze der OECD-Eurostat-Liste übernommen.

### Datengrundlagen und Analysen

Den vorgenommenen Analysen liegen Daten der Todesursachenstatistik sowie der Bevölkerungsstatistik zugrunde (Tab. 2).

**Table 2:** 

	Amtliche Todesursachenstatistik	Amtliche Bevölkerungsstatistik
Inhalte	Todesursachen, vierstellige ICD-Codes^a^ Äußere Ursachen von Morbidität und Mortalität (V01–Y84), vierstellige ICD-Codes^b^	Fortschreibung des Bevölkerungsstandes auf Basis des Zensus von 2011 Jahresmittelwerte berechnet aus den Stichtagswerten zum 31.12. zweier aufeinander folgender Jahre
Verwendete Kategorien und Jahre	Sterbealter nach Alterskategorien < 1 Jahr, 1–4 Jahre, 5–9 Jahre, …, 70–74 Jahre Getrennte Informationen für Männer und Frauen Informationen für Bayern sowie die einzelnen Regierungsbezirke Daten aus den Jahren 2016, 2017, 2018	Alterskategorien < 1 Jahr, 1–4 Jahre, 5–9 Jahre, …, 70–74 Jahre Getrennte Informationen für Männer und Frauen Informationen für Bayern sowie die einzelnen Regierungsbezirke Daten aus den Jahren 2016, 2017, 2018, 2019
Datenhalter/Zugang	Bayerisches Landesamt für Statistik Daten auf Anfrage	Bayerisches Landesamt für Statistik Daten frei zugänglich über GENESIS-online Bayern

^a^Komplette Mikrodaten, d. h., es wurden keine Daten zum Zwecke der Geheimhaltung entfernt oder umcodiert

^b^Für die Todesfälle, die einen vierstelligen ICD-Code aus den Kapiteln S–T haben, werden bei der Berechnung der vermeidbaren Sterblichkeit die dazugehörigen ICD-Codes der äußeren Ursache als die „todesursächlichen“ angesehen

Der Indikator zur vermeidbaren Sterblichkeit soll zum einen stabil gegenüber zufallsbedingten Schwankungen sein und zum anderen veränderungssensibel, d. h., er soll Veränderungen über die Zeit bzw. Unterschiede zwischen den Regionen aufzeigen. Dabei sollen neben Zufallseffekten auch systematische Verzerrungen möglichst vermieden werden. Zur Überprüfung der Veränderungssensibilität und Validität des Indikators werden die im Folgenden beschriebenen Analysen zur regionalen Variabilität sowie zu Veränderungen über die Zeit durchgeführt.

#### Analysen zur regionalen Variabilität.

Die Veränderungssensibilität des Indikators zwischen den Regierungsbezirken wird varianzanalytisch für das Jahr 2018 untersucht. Neben einer Betrachtung der Werte für die einzelnen Regierungsbezirke werden der Mittelwert über alle Regierungsbezirke sowie die Standardabweichung berechnet und diejenigen Regierungsbezirke ausgewiesen, die mehr als eine Standardabweichung über oder unter dem Durchschnitt liegen. Systematische regionale Unterschiede des Codierverhaltens sind grundsätzlich nicht anzunehmen, da die Signierkräfte in Bayern regionenübergreifend codieren.^2^ Dennoch wird anhand der Benutzung des ICD-Codes (Internationale Klassifikation der Krankheiten) für unbestimmte Todesursachen „R99“ („Sonstige ungenau oder nicht näher bezeichnete Todesursachen“ [23]) geprüft, ob es Hinweise auf regionale Codierunterschiede gibt. Eine niedrige Sterberate mit dem Code R99 ist ein Indikator für eine hohe Qualität der Todesursachenstatistik. Starke Unterschiede in der Verwendung dieses Codes könnten darauf hinweisen, dass nicht alle tatsächlich vermeidbaren Todesfälle in gleichem Maße in allen Regionen in den Indikator der vermeidbaren Sterblichkeit einfließen, was die Vergleichbarkeit über die Regionen einschränken würde. In der Analyse wurden hierfür die prozentualen Anteile an R99-Codes an allen Todesfällen im aktuellsten Jahr (2018) jeweils getrennt für Frauen und Männer und nach Regierungsbezirk untersucht.

#### Analysen der Veränderungen über die Zeit.

Um Einsichten in die zeitliche Variabilität bzw. Stabilität des Indikators zu gewinnen, werden die Werte der vermeidbaren Sterblichkeit für Bayern und die Regierungsbezirke für die Jahre 2016, 2017 und 2018 berechnet und Veränderungen über die Zeit grafisch visualisiert. Als zusätzlicher Anhaltspunkt für mögliche validitätskritische systematische Veränderungen über die Zeit wird untersucht, ob es über die Jahre 2016–2018 zu größeren Schwankungen des prozentualen Anteils der 10 wichtigsten Todesursachenkategorien der vermeidbaren Sterblichkeit bei Männern^3^ und Frauen^4^ kommt. Diese Schwankungen bzw. Verschiebungen in der Reihenfolge der Todesursachenkategorien könnten auf verzerrende Methodenbrüche hinweisen, wie eine Umstellung im Regelwerk, ein verändertes Codierverhalten oder ein verändertes Verhalten der Ärztinnen und Ärzte bei der Ausstellung der Todesbescheinigung.

## Ergebnisse

### Vermeidbare Sterblichkeit in Bayern

In Tab. 3 finden sich die berechneten Werte der vermeidbaren Sterblichkeit nach Geschlecht für das zum Zeitpunkt der Analyse aktuelle Jahr mit verfügbaren Daten (2018) für Bayern sowie eine Aufteilung nach prävenierbarer und durch Behandlung vermeidbarer Sterblichkeit.

**Table 3:** 

	Gesamtbevölkerung	Frauen	Männer
Sterbefälle vermeidbar durch Prävention	14,47	8,83	20,40
Sterbefälle vermeidbar durch Behandlung	8,48	7,30	9,77
Vermeidbare Sterbefälle gesamt	22,95	16,13	30,16

Es ergibt sich eine altersstandardisierte vermeidbare Sterblichkeit von knapp 23 Todesfällen je 10.000 Einwohnerinnen und Einwohner (EW) in Bayern im Jahr 2018, wobei die prävenierbaren Sterbefälle die behandelbaren bei Weitem überwiegen. Für Männer liegt die Sterberate aufgrund vermeidbarer Ursachen bei 30 pro 10.000 männlichen EW und ist damit fast doppelt so hoch wie jene für Frauen (16 pro 10.000 weiblichen EW). Vor allem bei den prävenierbaren Sterbefällen ist der Geschlechterunterschied sehr ausgeprägt. Der Unterschied in der vermeidbaren Sterblichkeit zwischen Frauen und Männern geht daher insbesondere auf Unterschiede bei den prävenierbaren Sterbefällen zurück.

### Vermeidbare Sterblichkeit in den bayerischen Regierungsbezirken

Tab. 4 zeigt die Ergebnisse zur vermeidbaren Sterblichkeit auf Ebene der Regierungsbezirke. Die Werte der Regierungsbezirke liegen zwar nahe am Durchschnittswert für Bayern, dennoch liegt eine gewisse Variation zwischen den Regierungsbezirken vor, die inhaltliche Rückschlüsse auf regionale Unterschiede der vermeidbaren Sterblichkeit erlaubt. Die höchste Sterberate aufgrund von vermeidbaren Ursachen für die Gesamtbevölkerung innerhalb Bayerns findet sich in Oberfranken (fast 27 pro 10.000 EW). Dieser Regierungsbezirk weist als einziger Bezirk eine vermeidbare Sterblichkeit bei Männern wie Frauen auf, die mehr als eine Standardabweichung entfernt vom Durchschnitt aller Regierungsbezirke liegt. Der niedrigste Wert für vermeidbare Sterblichkeit ergibt sich für Oberbayern (knapp 21 pro 10.000 EW).

**Table 4:** 

Regierungsbezirk	Gesamtbevölkerung	Frauen	Männer	Wert mehr als eine Standardabweichung entfernt vom Durchschnitt aller Regierungsbezirke
Oberbayern	20,77	15,22	26,84	Nein
Niederbayern	23,64	15,89	32,12	Nein
Oberpfalz	24,23	17,25	32,30	Nein
Oberfranken	26,92	18,32	35,91	Ja (Abweichung nach oben)
Mittelfranken	25,50	17,47	34,11	Nein
Unterfranken	22,17	15,92	28,87	Nein
Schwaben	22,14	15,43	29,23	Nein
*Bayern Gesamt*	*22,95*	*16,13*	*30,16*	–

Systematische regionale Unterschiede im Codierverhalten von Todesursachen sind, wie bereits erwähnt, nicht anzunehmen, da in Bayern die Codierkräfte regionenübergreifend arbeiten. Dies bestätigt auch die Überprüfung der Verwendung des ICD-Codes für unbestimmte Todesursachen R99 („Sonstige ungenau oder nicht näher bezeichnete Todesursachen“). Die Analyse des prozentualen Anteils an R99-Codes an allen Todesfällen im Jahr 2018 jeweils getrennt für Frauen und Männer und nach Regierungsbezirk zeigt, dass es nur eine geringe Variation der Nutzung des R99-Codes gibt. Für Frauen variiert der Prozentsatz über die Regierungsbezirke zwischen 0,24 % und 0,61 %, für Männer zwischen 0,33 % und 1,22 %.

### Veränderungen der vermeidbaren Sterblichkeit über die Zeit

**Figure 1:**
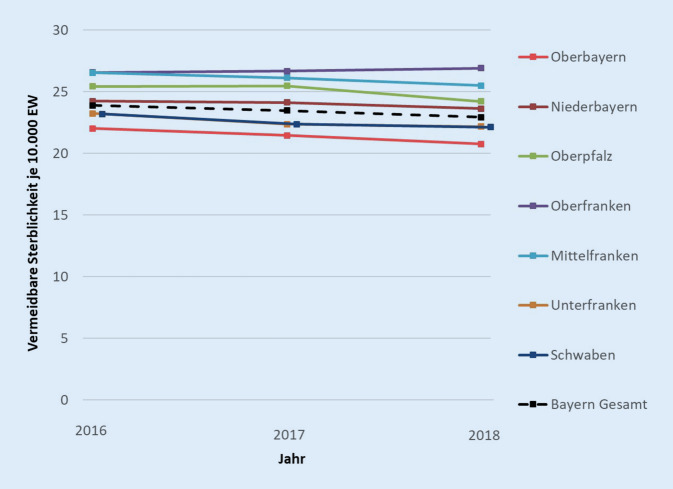

Die Ergebnisse der Zeitreihenanalyse zwischen 2016 und 2018 finden sich in Abb. 1.

Der Indikator ist demnach auf Bayern – wie auch auf Regierungsbezirksebene – sehr stabil über die Zeit und zeigt eine leichte Tendenz zur Verringerung der vermeidbaren Todesfälle. Darüber hinaus lassen sich aber auch unterschiedliche Entwicklungen für die Regierungsbezirke ausmachen: Während die vermeidbare Sterblichkeit in Oberfranken in den letzten 3 Jahren leicht angestiegen ist, ist sie in Oberbayern im selben Zeitraum zurückgegangen.

Die im Rahmen der Validitätsanalysen durchgeführte Überprüfung von Schwankungen für die 10 häufigsten Todesursachenkategorien der vermeidbaren Sterblichkeit für Männer und Frauen ergibt für beide Geschlechter sehr stabile Werte über die 3 Jahre von 2016 bis 2018 (detaillierte Ergebnisse nicht dargestellt). Dies betrifft sowohl jede Todesursache für sich betrachtet als auch den Gesamtanteil an allen vermeidbaren Sterbefällen. Für Männer lassen sich im Jahr 2018 mit diesen wichtigen Todesursachen 63 % aller vermeidbaren Sterbefälle abdecken, bei Frauen ca. 61 %.

## Diskussion und Fazit

Ziel der vorliegenden Arbeit war die Weiterentwicklung des Indikators der vermeidbaren Sterblichkeit für die Gesundheits- bzw. Präventionsberichterstattung im Anschluss an die internationale Diskussion sowie die Prüfung der Anwendbarkeit auf regionaler Ebene. Hierzu wurde auf den aktuellen, international konsentierten OECD-Eurostat-Indikator der vermeidbaren Sterblichkeit zurückgegriffen. Es zeigt sich, dass dieser Indikator im Sinne der eingangs gesetzten Ziele verwendet werden kann.

Im Vergleich zu den gegenwärtig in der Gesundheitsberichterstattung verwendeten Varianten des Indikators ist die Berechnung des neu gefassten Indikators zwar aufwendiger, vermittelt dafür jedoch ein umfassenderes und dem aktuellen Stand der Versorgung und Prävention angepasstes Bild des Ausmaßes der vermeidbaren Sterblichkeit und lässt erstmals eine Differenzierung in behandelbare und prävenierbare Sterblichkeit zu sowie Vergleiche auf regionaler wie auch internationaler Ebene. Die Daten aus Bayern und den Regierungsbezirken zeigen, dass die prävenierbaren Sterbefälle einen beträchtlich größeren Anteil an der vermeidbaren Sterblichkeit ausmachen als die behandelbaren Sterbefälle. Dies stellt ein wichtiges Ergebnis dar, auch vor dem Hintergrund des nach wie vor geringen Anteils der Präventionsausgaben an allen Gesundheitsausgaben, der in Bayern wie im übrigen Deutschland seit Jahren im Bereich zwischen 3–4 % liegt [25, 26].

Die empirische Überprüfung für Bayern und die bayerischen Regierungsbezirke ergibt, dass die errechneten Werte relativ stabil über die Zeit sind und damit nicht anfällig für größere zufallsbedingte Schwankungen erscheinen. Darüber hinaus könnten sich z. B. beim Ausfüllen der Todesbescheinigung systematische Unterschiede über die Zeit ergeben, wenn etwa eine im Zeitverlauf erhöhte Sensibilität für bestimmte Erkrankungen besteht, Änderungen im Regelwerk der Weltgesundheitsorganisation (WHO) oder im Signierverhalten der Fachkräfte in der amtlichen Statistik zu systematischen Verzerrungen führen. Die Untersuchungen zu zeitlichen Veränderungen bei den 10 wichtigsten Todesursachen der vermeidbaren Sterblichkeit liefern jedoch keinen Anlass, von derartigen Verzerrungen über die Zeit auszugehen.

Grundsätzlich können regional unterschiedliche Ergebnisse tatsächliche regionale Unterschiede bedeuten, auf eine regionenspezifische Codierpraxis zurückzuführen sein oder auch auf regionenspezifische Angaben der Ärztinnen und Ärzte in den Todesbescheinigungen. Eine regionenspezifische Codierpraxis konnte anhand der Prüfung der Verwendung des Codes R99 nicht nachgewiesen werden und ist aufgrund des regionenübergreifenden Signierens und durch die zunehmende Verwendung des elektronischen Codierkerns Iris/MUSE [24] nicht anzunehmen. Die beobachteten Unterschiede zwischen den Regierungsbezirken stehen zudem in Übereinstimmung mit früheren Untersuchungen zu Krankheitslast und Mortalität in Bayern und weisen ein Nord-Süd-Gefälle auf [27–29]. Es ist davon auszugehen, dass der Indikator belastbare regionale Vergleiche – auch zwischen den Bundesländern – ermöglicht. Eine empirische Prüfung letzterer Annahme steht jedoch aus.

Einschränkend ist festzuhalten, dass die hier durchgeführten Analysen keine Beurteilung der Qualität der Todesursachenstatistik einschließen. Für die Todesursachenstatistik werden der vertrauliche Teil der ärztlichen Todesbescheinigung und ggf. der Obduktionsschein von den Gesundheitsämtern an die statistischen Landesämter übermittelt. Dort wird auf Basis des Regelwerkes der WHO das sogenannte Grundleiden ermittelt, also „a) die Krankheit oder Verletzung, die den Ablauf der direkt zum Tode führenden Krankheitszustände auslöste, oder b) die Umstände des Unfalls, oder der Gewalteinwirkung, die den tödlichen Ausgang verursachten“ [23]. Die Qualität der Todesbescheinigungen und der Leichenschau wie auch die Erfassung und unikausale Signierung der Todesursachen bei der Erstellung der Todesursachenstatistik sind seit Langem Gegenstand kritischer Diskussionen (siehe zuletzt das Schwerpunktheft 12/2019 des Bundesgesundheitsblatts). So wird es zum Beispiel aufgrund der mit zunehmendem Alter ansteigenden Komorbidität für Ärztinnen und Ärzte schwieriger, beim Ausstellen der Todesbescheinigungen eine schlüssige und plausible Kausalkette anzugeben, die zum Tode geführt hat, womit die eindeutige Codierung einer einzelnen zugrunde liegenden Todesursache deutlich erschwert wird [30]. Die daraus resultierenden Probleme betreffen alle Auswertungen der Todesursachenstatistik und sollten bei der Interpretation der Befunde mitbedacht werden.

Zu beachten ist, dass die Aussagekraft des Indikators abnimmt, je kleinräumiger die betrachteten Regionen sind. Auf Kreisebene muss mit erheblichen Einschränkungen der Aussagekraft gerechnet werden, insbesondere aufgrund der kleinen Fallzahlen für einzelne Todesursachen und der hier stärkeren Einflüsse des Dokumentationsverhaltens einzelner Ärztinnen und Ärzte bei der Leichenschau. Zudem lässt der Indikator auf kleinräumiger Ebene nur bedingt Aussagen über das örtliche Präventions- und Versorgungssystem zu. Er macht zunächst nur eine Aussage über die vermeidbaren Sterbefälle der Bevölkerung am Wohnort, ohne deren Ursachen zu lokalisieren. Mit Ausnahme von Unfällen, Gewalttaten und Suiziden sind die Ursachen vermeidbarer Sterbefälle über die Todesursachenstatistik meist nicht örtlich lokalisierbar. Schlussfolgerungen aus den Daten für die Weiterentwicklung der regionalen Prävention bzw. des regionalen Versorgungssystems erfordern daher die Hinzuziehung weiterer Daten.

Der Indikator zur vermeidbaren Sterblichkeit ist wie z. B. die (gesunde) Lebenserwartung ein Globalindikator, der mehrdimensional und auf hoher Aggregationsebene misst, was nicht von einzelnen Indikatoren erfasst werden kann [31]. Als Folge des hohen Aggregationsgrades des Indikators benötigen auffällige Signale allerdings weiterführende Analysen und eine kontextbezogene Interpretation. Im Rahmen der vorliegenden Arbeit werden zwar keine vertiefenden Analysen zur Interpretation der Unterschiede der vermeidbaren Sterblichkeit zwischen den Regierungsbezirken vorgenommen. Allerdings sind die Befunde auf Regierungsbezirksebene, wie bereits erwähnt, konsistent mit früheren Regionalanalysen zu Gesundheitsverhalten und Sterblichkeit in Bayern. Auch die Unterschiede der vermeidbaren Sterblichkeit nach Geschlecht sind vor dem Hintergrund der allgemein höheren Lebenserwartung bei Frauen in Deutschland und der lebensstilassoziierten Risikofaktoren einer vorzeitigen Sterblichkeit bei Männern gut interpretierbar. Zu ähnlichen Rückschlüssen kommen auch frühere Studien zu kleinräumigen Unterschieden der vermeidbaren Sterblichkeit in Deutschland [16, 32].

Die vorliegende Analyse zeigt somit, dass der Indikator mit der OECD-Eurostat-Liste auch regional umsetzbar ist und plausible Befunde zur regionalen Gesundheit liefert. Die gegenüber seinen Vorläufern komplexere Konstruktion kann programmiert werden und stellt kein Anwendungshindernis dar. Daher sollte seine Aufnahme in die Routine der Gesundheits- bzw. Präventionsberichterstattung geprüft werden. Sinnvoll wäre eine regelmäßige Reevaluation und ggf. Anpassung der Todesursachenliste nach 10 oder 15 Jahren unter Berücksichtigung der zwischenzeitlichen Entwicklungen in Prävention und Gesundheitsversorgung. Die Reevaluation der Todesursachen könnte in enger Abstimmung mit Entwicklungen der internationalen OECD-Eurostat-Liste vorgenommen werden.

## Footnotes

1 Todesursachen, welche in einer der beiden Listen verzeichnet sind, sind auch in der OECD-Eurostat-Liste enthalten. Die einzige Ausnahme sind die folgenden Todesursachen aus dem Bereich der Lebererkrankungen, welche zusätzlich in der AOLG-Liste enthalten sind, aber nicht in der OECD-Eurostat-Liste: K71, K72, K74.3–K74.5, K75, K76, K77. Diese Todesursachen machten allerdings nur 0,17 % aller Todesursachen im Jahr 2017 aus [21, 22] und können somit als vernachlässigbar angesehen werden. Laut OECD sind sehr seltene Todesursachen mit sehr kleinen Todesfallzahlen nicht in der Liste enthalten, damit die Liste so übersichtlich wie möglich bleibt [15].

2 Zudem wurde in den Jahren 2016 bis 2018 in Bayern der elektronische Codierkern Iris/MUSE (Iris Institut/BfArM/Destatis, Bonn, Deutschland) [24] als Signierhilfe genutzt. Dies trug zu einer zusätzlichen Vereinheitlichung des Signierprozesses bei. Seit 2020 ist die Software in Bayern vollumfänglich im Einsatz.

3 Diese sind in absteigender Reihenfolge der Häufigkeit: ischämische Herzkrankheiten, bösartige Neubildung der Trachea, der Bronchien und der Lunge, alkoholbedingte Krankheiten: alkoholspezifische Störungen und Vergiftungen, zerebrovaskuläre Krankheiten, bösartige Neubildung des Kolons, am Rektosigmoid, des Rektums und des Anus, absichtliche Selbstverletzung, Unfallverletzungen, Diabetes mellitus, Transportmittelunfälle, Hypertonie (Hochdruckkrankheit).

4 Diese sind in absteigender Reihenfolge der Häufigkeit: bösartige Neubildungen der Brustdrüse, bösartige Neubildung der Trachea, der Bronchien und der Lunge, ischämische Herzkrankheiten, zerebrovaskuläre Krankheiten, bösartige Neubildung des Kolons, am Rektosigmoid, des Rektums und des Anus, alkoholbedingte Krankheiten: alkoholspezifische Störungen und Vergiftungen, Unfallverletzungen, absichtliche Selbstverletzung, Diabetes mellitus, Hypertonie (Hochdruckkrankheit).
